# Blocking the Interactions between Calcium-Bound S100A12 Protein and the V Domain of RAGE Using Tranilast

**DOI:** 10.1371/journal.pone.0162000

**Published:** 2016-09-06

**Authors:** Jian Wei Chiou, Brian Fu, Ruey-Hwang Chou, Chin Yu

**Affiliations:** 1 Department of Chemistry, National Tsing Hua University, Hsinchu, 30013, Taiwan; 2 Northwood High School, Irvine, California, 92620, United States of America; 3 Graduate Institute of Cancer Biology and Center for Molecular Medicine, China Medical University, Taichung, 40402, Taiwan; 4 Department of Biotechnology, Asia University, Taichung, 41354, Taiwan; Russian Academy of Medical Sciences, RUSSIAN FEDERATION

## Abstract

The receptor for advanced glycation end products (RAGE), a transmembrane receptor in the immunoglobulin superfamily, is involved in several inflammatory processes. RAGE induces cellular signaling pathways upon binding with various ligands, such as advanced glycation end products (AGEs), β-amyloids, and S100 proteins. The solution structure of S100A12 and the V ligand-binding region of RAGE have been reported previously. Using heteronuclear NMR spectroscopy to conduct ^1^H–^15^N heteronuclear single quantum coherence (HSQC) titration experiments, we identified and mapped the binding interface between S100A12 and the V domain of RAGE. The NMR chemical shift data were used as the constraints for the High Ambiguity Driven biomolecular DOCKing (HADDOCK) calculation to generate a structural model of the S100A12–V domain complex. In addition, tranilast (an anti-allergic drug) showed strong interaction with S100A12 in the ^1^H–^15^N HSQC titration, fluorescence experiments, and WST-1 assay. The results also indicated that tranilast was located at the binding site between S100A12 and the V domain, blocking interaction between these two proteins. Our results provide the mechanistic details for a structural model and reveal a potential precursor for an inhibitor for pro-inflammatory diseases, which could be useful for the development of new drugs.

## Introduction

The authors of several studies have reported that members of the S100 protein family play regulatory roles in cells, and induce cell growth and differentiation [1, 2]. S100 proteins are small, with molecular weights of approximately 10–13 kDa. Human S100 calcium-bound protein (S100A12) was first identified in neutrophil cells and is expressed primarily in granulocytes [3, 4]. Previous studies have indicated that members of the S100 protein family play important roles in tumor progression, so these proteins are commonly used as tumor markers [5]. The human S100A12 protein is overexpressed in several tissues in conditions such as gastric carcinoma, diabetes, Crohn’s disease, and Mooren’s ulcer. These diseases are usually related to the inflammation of cells [6–9]. The S100A12 protein expresses its bio-activity after calcium ions bind to its EF-hand domains [10–12]. S100A12 has different structural states that lead to different biological functions, and these states are caused by the presence of different metal ions [13, 14]. Calcium-binding proteins such as S100A12 expose specific ligand-binding sites, which activate cell signaling pathways such as MAPK, NF-κB, and ERK. Therefore, S100A12 is recognized as an important biomarker for detecting cancer [15–18].

The receptor for advanced glycation end products (RAGE) is a multi-ligand cell surface receptor that consists of three parts: an extracellular domain, a transmembrane domain, and a cytoplasmic domain. The extracellular domain belongs to the immunoglobulin superfamily [19, 20]. The V, C1, and C2 domains of the extracellular domain usually bind with various ligands including the high-mobility group box 1 (HMGB1) protein, advanced glycation end products (AGEs), transthyretin, DNA, and β-amyloids [21–25]. This binding between RAGE and ligands can activate mitogen-activated protein (MAP) kinases such as JNK, MAPK, p38, and p44/42 [26–28]. The authors of several studies have reported that the interactions between RAGE and S100 proteins are the cause of many disorders [29]. Such interactions induce signal transduction through the transmembrane domain and cause the phosphorylation of the cytoplasmic domain, which then activates certain signaling pathways *in vivo* [30]. The chain of signaling cascades results in cell growth, proliferation, tumor generation, and neurite outgrowth, and causes some inflammatory-related diseases [31–33].

Recently, RAGE has become an important therapeutic target because it is associated with a variety of human diseases as well as tumor growth [34–36]. To better understand the mechanism of RAGE–ligand binding, we generated a structural model using heteronuclear NMR spectroscopy and High Ambiguity Driven biomolecular DOCKing (HADDOCK) structural calculations [37]. Structural studies have been carried out on the binding between certain S100 proteins and RAGE, and on the subsequent complexes formed including mutant (C3S) S100A6–RAGE V [38], S100P–RAGE V [39], S100A11–RAGE V [40], and S100A12–RAGE C1C2 [41]. These studies revealed that the RAGE V domain binds to the region around helix 4 of the S100 proteins. However, the RAGE V domain-binding site differs among S100 proteins. These discrepancies may be caused by differences in the net charge, polarity, amino acid sequence, or other properties of the S100 proteins. The study on S100A12– RAGE C1C2 also demonstrated the interaction between RAGE C1C2 and the S100A12 surface. However, the nature of the binding region in the RAGE C1C2 domain remains unclear.

Moroz *et al*. demonstrated that the oligomeric state of S100A12 requires the presence of zinc ions [42]. This suggests that the S100A12 protein is a dimer at certain calcium concentrations (1–10 mM calcium ion). In the absence of relevant structural studies, the nature of the interaction between the S100A12 dimer and RAGE remains unclear. Leclerc *et al*. (2009) have described how surface plasmon resonance (SPR) reveals that the V domain of RAGE binds to S100A12 [43]. Here, we report the structure of the complex formed by the S100A12 dimer and the V domain of RAGE. Furthermore, we found that tranilast (an anti-allergic drug; please see S1 Fig for the structure) [44, 45] efficiently inhibited the cell proliferation caused by S100A12–RAGE V signaling transduction, indicating that it may be a potential precursor for a therapeutic inhibitor. These results provide structural insight into the activation of RAGE by S100A12, and reveal a useful precursor for new drug development.

## Materials and Methods

### 2.1 Materials

Luria broth was purchased from Amresco. ^15^NH_4_Cl and D_2_O were purchased from Cambridge Isotope Laboratories. Tranilast was purchased from Sigma. The SW-480 cell line was obtained from the American Type Culture Collection (CCL-288). The cDNA of S100A12 and the RAGE V domain were purchased from Mission Biotech Company using vectors pET21b for S100A12 and pET-32b (+) for the RAGE V domain. The genes were subcloned into *Escherichia coli BL21* (DE3) (Novagen). The details of the purification process for obtaining the pure S100A12 and the RAGE V domain proteins are given in the Supporting Information. The cell proliferation reagent WST-1 (4-[3-(4-iodophenyl)-2-(4-nitrophenyl)-2H-5-tetrazolio]-1,3-benzene disulfonate) was purchased from Roche. FPS-ZM1, an inhibitor of the RAGE V domain, was purchased from Calbiochem [46].

### 2.2 NMR HSQC titration experiments

The HSQC spectra was collected using a Varian 700 MHz NMR spectrometer with cryogenic probes at 298 K. All titrations were carried out using the same buffer composition (20 mM Tris-HCl, 5 mM CaCl_2_, 100 mM NaCl, 10% D_2_O, pH 7). Hung *et al*. have made available the backbone and side-chain assignments for S100A12 in specific buffer conditions (10 mM Hepes, 100 mM NaCl, and 0.02% (w/v) NaN_3_, pH 6.5) at the Biological Magnetic Resonance Bank (BMRB) (BMRB code: 19293) [47], and the assignments for the RAGE V domain in specific buffer conditions (20 mM sodium phosphate, pH 7.5, and 100 mM Na_2_SO_4_) have been reported by Matsumoto *et al*. (BMRB code: 7364) [48]. We used three-dimensional spectra including those of HNCA, HNCOCA, HNCACB, and CBCACONH to compare the cross-peak assignment in our study with the assignments reported by Hung and Matsumoto.

The titration experiment was performed by adding unlabeled calcium-binding S100A12 to the ^15^N-labeled RAGE V domain solution at molar ratios of 1:0, 1:0.33, 1:0.66, 1:1, and 1:2. The reverse titration (^15^N-labeled S100A12 with the addition of unlabeled RAGE V domain) was performed at molar ratios of 1:0, 1:0.25, 1:0.5, 1:0.75, and 1:1. Finally, the titration experiment with ^15^N-labeled S100A12 and tranilast was performed at molar ratios of 1:0, 1:0.5, 1:1, and 1:2. By overlaying the HSQC spectra at different ratios, we identified certain residues at the interface of the two molecules that had decreased intensity or perturbed chemical shift.

### 2.3 Molecular docking

HADDOCK is useful software for calculating protein–protein docking. We used it to obtain the complex structures of the S100A12–RAGE V domain and S100A12–tranilast complexes. The structural coordinates of calcium-binding S100A12 were taken from the Protein Data Bank (PDB ID: 2m9g) [47]. The structural coordinates of the RAGE V domain were also taken from the Protein Data Bank (PDB ID: 2e5e) [48]. The input data for tranilast were obtained from the DrugBank database (accession number: DB07615). The residues with obvious perturbations or decreased intensity were identified by bar diagrams and defined as ambiguous interaction constraints for the residues at the interface of S100A12 and the RAGE V domain in the HADDOCK calculation [49]. All residues were defined by NACCESS [50] to divide them into active or passive categories in the input data for the HADDOCK program. The first calculation contained 2,000 total structures from rigid-body docking using the standard HADDOCK protocol with optimized potential for liquid simulation (OPLSX) parameters. We used the 200 lowest energy structures for the subsequent semi-flexible simulated annealing process to optimize the side-chain contacts by explicit solvent refinement. PyMOL [51] was used for all data for structural representations.

### 2.4 Fluorescence experiments to determine the binding constant (K_d_) between two proteins

Fluorescence titration can be a useful method for measuring the binding affinity of a protein–ligand interaction [52–54]. We used a Hitachi F-2500 fluorescence spectroscope to perform the florescence experiments. There is no tryptophan residue in S100A12; however, there is one tryptophan in the RAGE V domain that is exposed in the presence of a solvent. Therefore, we utilized the absorption band of tryptophan, which is found at a wavelength of 295 nm. We observed an emission curve in the range of 310 nm to 420 nm. The S100A12 protein was added to the RAGE V domain solution, which had a concentration of approximately 1.5 μM. Changes in the emission spectrum were monitored as the total concentration of S100A12 in the complex solution was increased (0 μM to 3.3 μM). We plotted the data as [S100A12] versus (I − I_0_). The program used the following equation [55] to calculate the extent of binding from the original curve:

$$\frac{1}{\left(I-I_{0}\right)}=\frac{1}{\left(I_{1}-I_{0}\right)}+\frac{K_{d}}{\left(I_{1}-I_{0}\right)}\times \frac{1}{\left[S100A12\right]}$$ (1)

In Eq (1), I_0_ represents the fluorescence intensity of the solution in the absence of S100A12; I represents the fluorescence intensity of the solution that contained S100A12 and the RAGE V domain; I_1_ represents the fluorescence intensity at the end of the experiment; and K_d_ represents the dissociation constant. The original curve was further processed by fitting to a linear curve and the slope was calculated to obtain the dissociation constant.

A similar approach was used with tranilast. The structure of tranilast contains a benzyl group, which exhibits obvious emission upon excitation at 333 nm. Tranilast exhibited a broad absorption peak at 335 nm in the UV spectra. By monitoring S100A12 at different concentrations (0–7.5 μM) in solution with tranilast (at a concentration of approximately 2.5 μM) and using an excitation wavelength of 333 nm, we obtained a curve and determined the extent of binding between S100A12 and tranilast.

### 2.5 Functional *in vitro* study using a WST-1 assay

Previous studies have shown that binding between S100A12 and RAGE activates the NF-κB signaling pathway [56], which is related to the survival rate and proliferation of cancer cells [57]. Therefore, we used cell proliferation as an index for the biological functions that respond to the downstream effects of the interactions between exogenous human S100A12 and the RAGE V domain. The WST-1 molecule is a tetrazolium salt that can be reduced to formazan by reductase in the mitochondria within cells [58, 59]. With the expansion of viable cells (cell proliferation), the activity of mitochondrial dehydrogenases increases, leading to an increase in the amount of formazan. Thus, the change in the optical density (OD) value at the appropriate wavelength is directly related to the number of metabolically active cells in the culture.

In this study, a WST-1 cell proliferation assay was performed according to the manual (Roche). The cells were cultured to the logarithmic growth phase, trypsinized, and seeded at a density of 1 × 10^4^ cells/well in a 96-well plate on the day before experiments. Subsequently, the cells were incubated in a serum-free medium containing 0.1% bovine serum albumin (BSA) for 24 h, after which the proteins (10, 50 or 100 nM S100A12, or 1 μM RAGE V domain) or drugs (1 μM tranilast or 1 μM FPS-ZM1) were added to the serum-starved cells, which were incubated for a further 48 h. Before harvest, 1/10 volume of WST-1 was added to each well and the cells were incubated at 37°C for a further 4 h. The medium in the cell culture plate was mixed by gentle agitation on a shaker for 10 min. The absorbance was measured at 450 nm using a Synergy 2 microplate reader (BioTek Instruments, Inc.). The relative cell numbers were determined by the absorbance relative to that observed in the control [60, 61].

## Results

### 3.1 Mapping the binding sites between S100A12 and the RAGE V domain

Using a two-dimensional HSQC spectrum, we conducted the titration experiments with the assignments reported previously by Hung *et al*. (S100A12) and Matsumoto *et al*. (RAGE V) to map the binding region. An overlay of the spectra of the ^15^N- labeled S100A12 alone and the ^15^N-labeled S100A12 complex with the unlabeled RAGE V domain showed that the signals of these residues changed (either the signal was perturbed or the intensity decreased) as the protein complex formed. The perturbation and decreased intensity originated from the residues at the interface between S100A12 and the RAGE V domain. To determine the extent of the changes in the HSQC cross-peaks, we recorded the chemical shift of the cross-peaks in two different dimensions, ^1^H and ^15^N, in the spectra at a protein–ligand ratio of 1:0 (H_0_, N_0_) and the complex spectra (H_1_, N_1_) at a ratio of 1:1. Next, we calculated the extent of perturbation using the following formula [62]:

$$Chemical\;shift\;difference=\sqrt{{\left(∆H\right)}^{2}+{\left(\frac{∆N}{6.51}\right)}^{2}}$$ (2)

**Fig 1:**
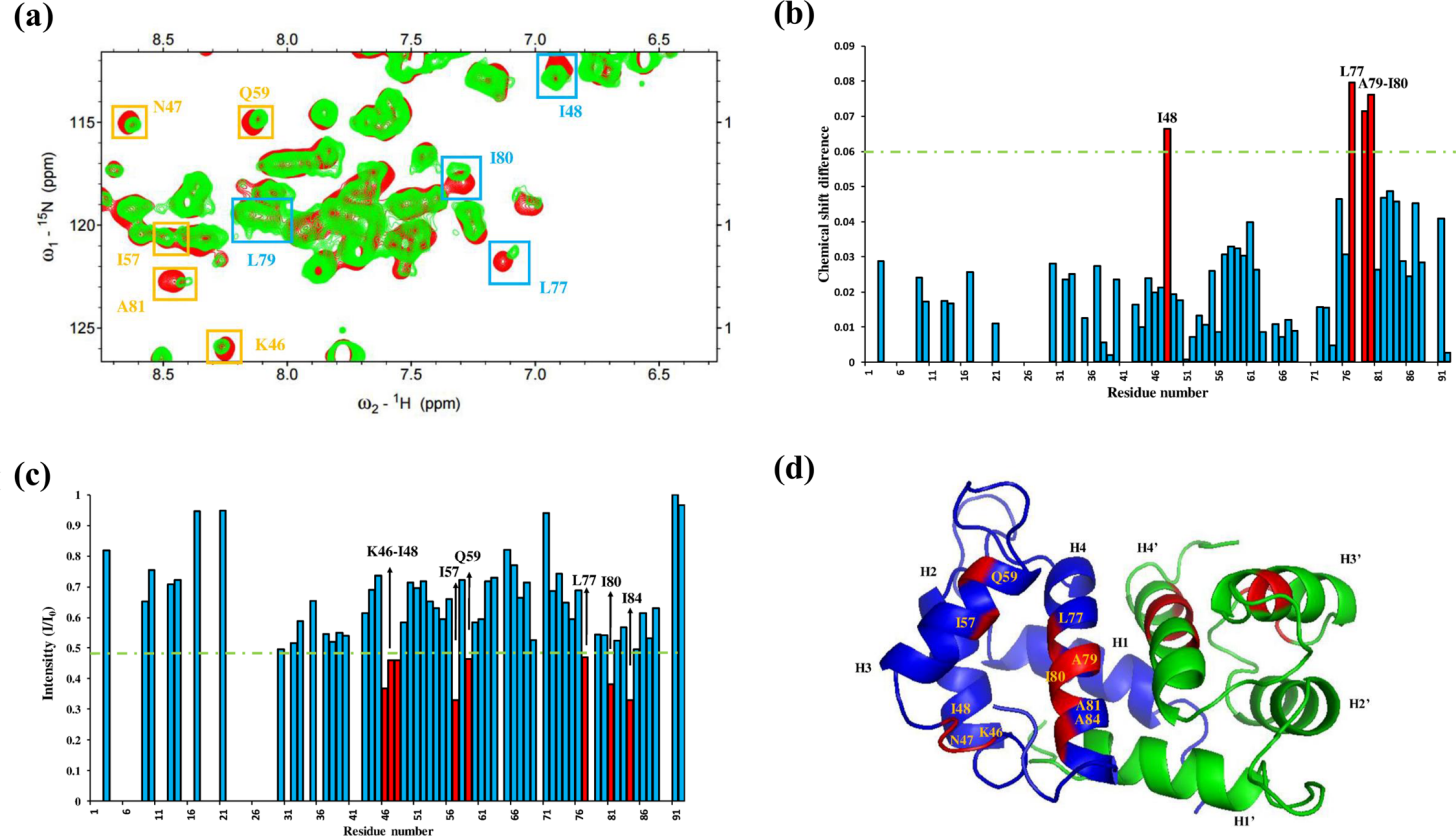
Analysis of the ^1^H–^15^N HSQC spectra of S100A12 in complex with the unlabeled RAGE V domain. (a) Overlay of the ^1^H–^15^N HSQC spectra of 0.76 mM ^15^N-labeled S100A12 (red) and S100A12 in complex with 0.76 mM unlabeled RAGE V domain (green). The residues that changed are indicated by yellow (decreased intensity) and cyan (perturbation) boxes, and were identified using bar diagrams. (b) Bar graph of cross-peak chemical shift perturbation where the green line represents the threshold of selected residues exhibiting significant changes. (c) Bar graph of cross-peak intensity (I/I_0_), where I represents the cross-peak intensity of the spectra of the complex solution (S100A12–RAGE V domain) and I_0_ is the cross-peak intensity of the spectra of S100A12 alone. (d) Ribbon representation of S100A12, the residues exhibiting significant changes are marked in red. H1 to H4 indicate helix 1 to helix 4 in the S100A12 monomer.

Fig 1A shows the overlay of the following two ^1^H–^15^N HSQC spectra: (1) ^15^N- labeled S100A12 alone and (2) ^15^N- labeled S100A12 with an unlabeled RAGE V domain.

We recorded the cross-peak intensity in the HSQC spectrum of labeled S100A12 alone (I_0_) and the intensity of the same cross-peak in the spectrum of S100A12 (labeled) bound with the unlabeled RAGE V domain (I_1_). To identify the residues involved in S100A12 in complex with the RAGE V domain, we plotted bar diagrams to compare the titration results, and identified cross-peak perturbation (Fig 1B) and decreased intensity (Fig 1C). The residues K46, N47, I48, I57, Q59, L77, A79, and I80 formed a hydrophobic region located at the linker region (residues 41–50), helix 3 (residues 51–61), and helix 4 (residues 71–85). These residues are depicted in red in the NMR structure of S100A12 from the Protein Data Bank (PDB ID: 2m9g), as shown in Fig 1D.

**Fig 2:**
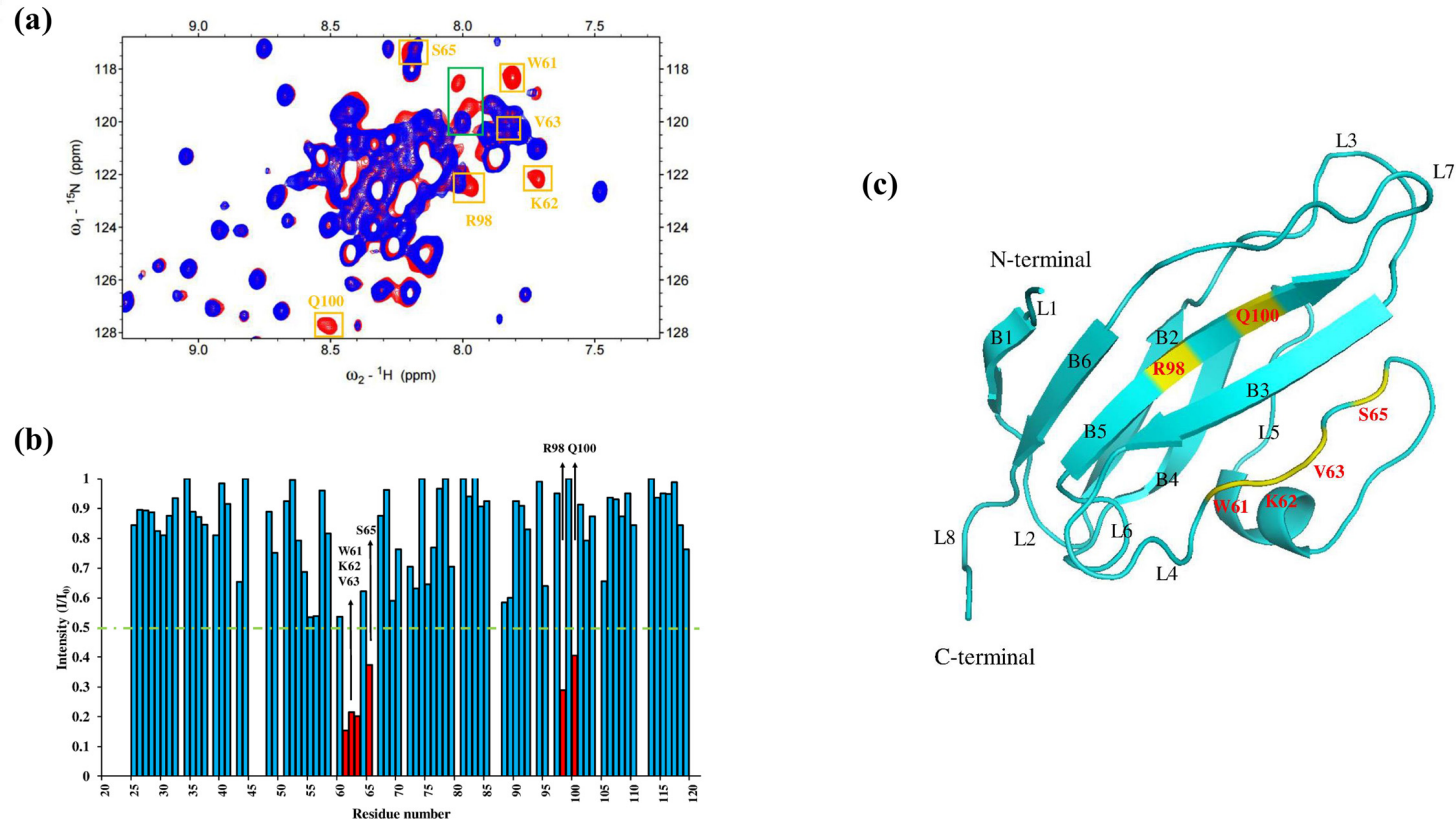
Analysis of the ^1^H–^15^N HSQC spectrum of the labeled RAGE V domain with unlabeled S100A12. (a) Overlay of the ^1^H–^15^N HSQC spectra of 0.5 mM ^15^N-labeled RAGE V domain (red) and the spectra of the complex with 0.5 mM unlabeled S100A12 (blue). The only changes were decreases in the cross-peak intensity, which are marked as yellow boxes. Two residues (green boxes) exhibited obvious changes but lacked assignment. (b) Diagram of the cross-peak intensity ratio (I/I_0_), where I represents cross-peak intensity in the spectra of the complex and I_0_ represents the cross-peak intensity in the spectra of the RAGE V domain alone. The green line represents the threshold for selecting residues that showed obvious decreases in intensity, and the selected residues are shown in red. (c) Ribbon representation of the RAGE V domain, in which the selected residues are mapped (yellow). B1–B6 correspond to the beta sheet and L1–L8 refer to the loop in the RAGE V domain.

The results of the ^1^H–^15^N HSQC titration experiment using the labeled RAGE V domain with unlabeled S100A12 revealed the six residues (W61, K62, V63, S65, R98, and Q100) in the binding interface (Fig 2A). Most of the interaction residues are located in loop 4, which is a flexible region. The overlaid HSQC spectra of the titration results and the bar diagram indicating decreased intensity are shown in Fig 2B. The NMR structure of the RAGE V domain (PDB ID: 2e5e) was determined by Matsumoto *et al*. [48], as shown in Fig 2C. The residues exhibiting decreased intensity (Fig 2A, red bar) reflect the binding sites on the RAGE V domain that bind to S100A12. These residues are shown in yellow in Fig 2C.

### 3.2 Model of the S100A12–RAGE V domain complex structure

The binding sites mapped using the results of the NMR titration experiment represent a region of S100A12 that forms a molecular complex with the RAGE V domain. We generated a model of the protein complex to characterize the protein–protein interactions using HADDOCK. The interaction residues exhibiting an intensity change or a chemical shift perturbation in the ^15^N-HSQC titration experiments were chosen as the input constraints for the HADDOCK calculation. Most of these residues were close to each other and formed a region of binding sites between S100A12 and the RAGE V domain. We first set up the HADDOCK calculation using 2,000 complex structures and used rigid-body minimization. The program then chose the best 400 structures by minimizing the total energy. Finally, further analysis of the torsion angle and subsequent Cartesian dynamics were performed in a solvent (water) model to calculate the best site for protein binding. The final results generated the best 200 structures and divided them into a single cluster. We took the best 10 structures from the cluster and the details of the calculation are shown in Table 1.

**Table 1:** Results of 10 best S100A12-RAGEV structure using HADDOCK.

Parameter	Value
**NMR restraint**	
Total NOE restraints	50
Total unambiguous NOE restraints	10
Ambiguous interaction restraints (AIR)	40
**CNS energy [kcal/mol] (after water refinement)**	
E_tot_	-786.3±27.1
E_vdw_	-123.1±16.2
E_electr_	-663.1±50.8
**Violation**s(dihedral violations)	
Violation >5°	0.0±0.0
Violation >10°	0.0±0.0
**RMSD for idealized geometry of best 200 structure**	
Bond(Å)	0.00292±0.00004
Angle (°)	0.428±0.006
RMSD for the best 200 structure	3.86
RMSD for the best 10 structure	2.85
**PROCHECK analysis**	
Residues in most favored regions (%)	80.5
Residues in addition allowed regions (%)	16.8
Residues in generously allowed regions (%)	1.5
Residues in disallowed regions (%)	1.2

**Fig 3:**
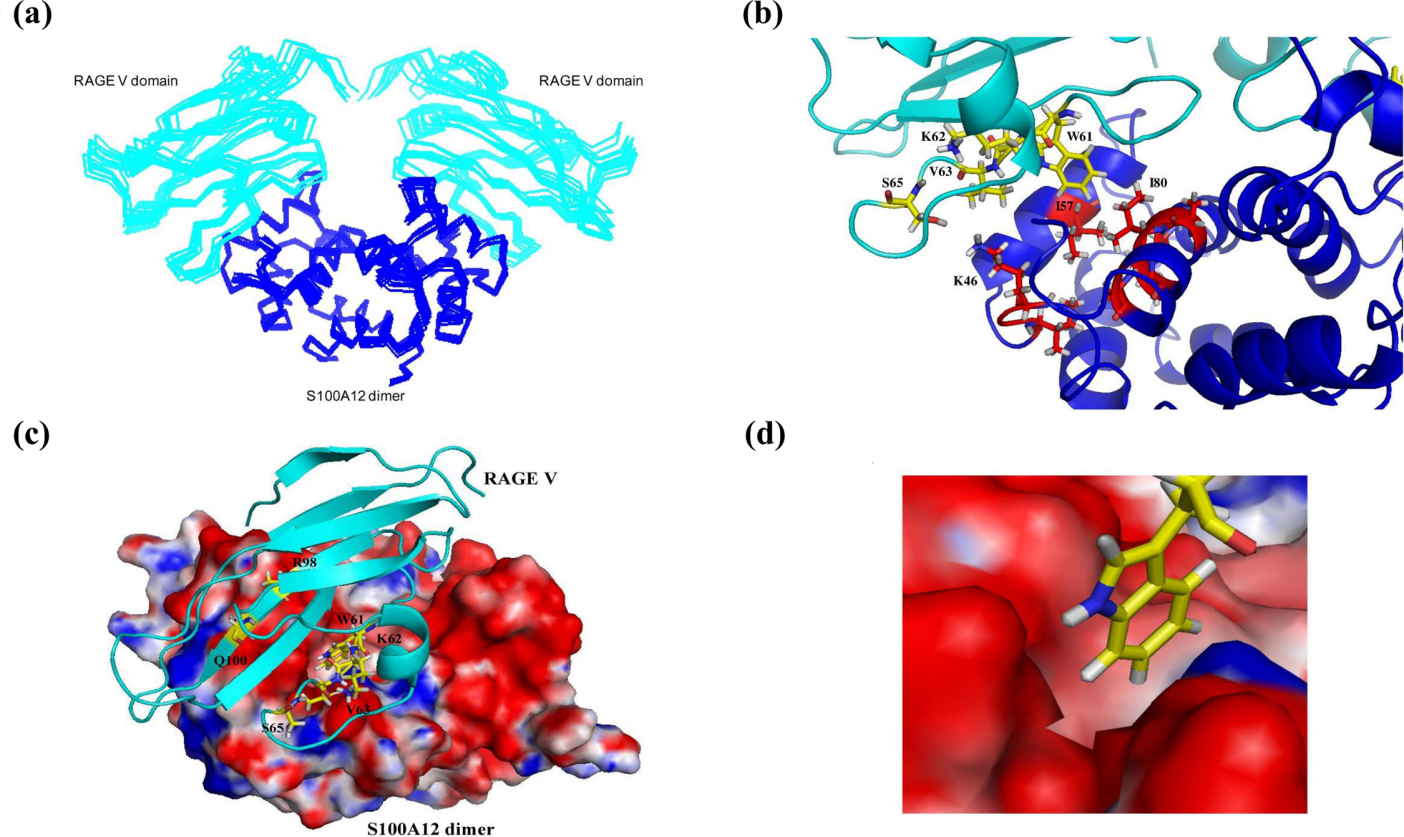
**The binding model of RAGE V domain and S100A12** (a) The ensemble solution structures of the S100A12–RAGE V domain binary complex overlaid after the HADDOCK calculation. (b) The binding sites of the S100A12–RAGE V domain complex. Ribbon representation of the binding region of S100A12 (blue ribbon) in complex with the RAGE V domain (cyan ribbon). The residues involved in the interaction are represented as red (S100A12) and yellow (RAGE V domain) sticks. (c) Electrostatic surface representation of the binding interface of S100A12 with the RAGE V domain (cyan ribbon). The positive region is colored blue and the negative region is red. (d) Expanded picture showing the binding region of W61 of RAGE V with the S100A12 surface. The atoms in S100A12 and the RAGE V domain are colored gray (protons), red or yellow (carbon atoms), and blue (nitrogen atoms).

The binding sites between S100A12 and the RAGE V domain are shown in Fig 3. The 10 best structures of the S100A12-RAGE V domain complex obtained from HADDOCK are shown in Fig 3A. The hydrophobic residues I57 and I80 of S100A12 interacted with the hydrophobic residue W61 of the RAGE V domain, as shown in Fig 3B. The hydrophilic residue K46 of S100A12 interacted with S65 of the RAGE V domain (Fig 3B). The residues Q59 of S100A12, and R98 and Q100 of RAGE V were remote from the other two binding interfaces (Fig 3C). The chemical shift change in these residues may have been caused by a slight conformational change. The results also show the W61 residue of RAGE V located in the pocket of the S100A12 surface (Fig 3D). Analysis of the average S100A12-RAGE V structure was carried out using the PROCHECK program [63]; the results indicate a reasonable stereochemistry for the structure. In the partition of the Ramachandran plot statistics, the results showed only 1.2% residues in the disallowed region and an overall average G-Factor of 0.1. These results indicate that the average structure was the usual region (S1 Table).

### 3.3 Mapping the binding sites between tranilast and S100A12 using ^1^H–^15^N HSQC titration experiments

**Fig 4:**
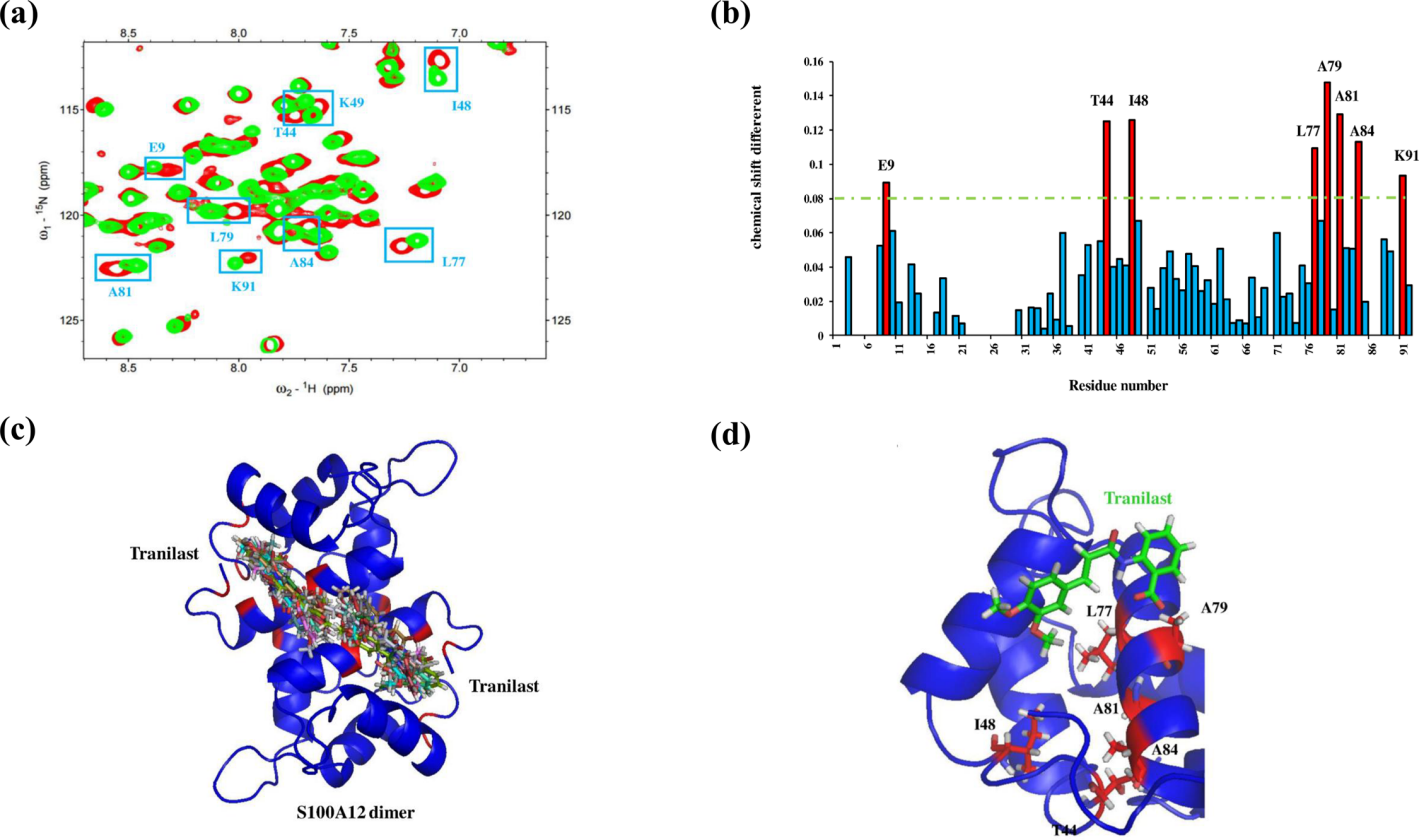
Analysis of the ^1^H–^15^N HSQC spectra of the labeled S100A12 in complex with the ligand (tranilast). (a) Overlay of the ^1^H–^15^N HSQC spectra of 0.5 mM ^15^N-labeled S100A12 (red) and S100A12 in complex with 0.5 mM tranilast (green). The results only indicate chemical shift changes and the cross-peaks are marked as cyan boxes. (b) Bar diagram of the cross-peak chemical shift perturbation plotted using HSQC titration data. The green line represents the threshold of selected residues that showed obvious changes in chemical shift, and the selected residues are shown in red. (c) Overlay of the lowest energy conformations of the clusters obtained from the HADDOCK calculation showing the binding region of tranilast. (d) Ribbon representation of S100A12, with the selected residues marked in red. Tranilast is shown in green, and the atoms in S100A12 and tranilast are colored gray (protons), red or green (carbon atoms), pink (oxygen atoms), and blue (nitrogen atoms).

The ligand–protein interaction between tranilast and S100A12 can also be identified by ^1^H–^15^N HSQC titration experiments. The ^1^H–^15^N HSQC spectra of S100A12 alone and with tranilast were compared (as shown in Fig 4A), and the cross-peaks exhibiting chemical shift perturbations were plotted as bar diagrams, as shown in Fig 4B. The residues that exhibited significant changes of chemical shift were E9, T44, I48, K49, L77, A79, A81, K84, and K91 (Fig 4A and 4B). The results indicate that some residues in this interface, such as I48, L77, A79, and A81, were also present in the interface between S100A12 and RAGE V. For the structural calculation by HADDOCK, the input data for the ambiguous interaction restraints (AIRs) were defined from the residues with obvious chemical shift perturbation in the HSQC titration experiments. The HADDOCK calculation provided the 200 structures of solvent (water) refinement. We chose the 10 best structures from the 200, as shown in Fig 4C. Tranilast is a hydrophobic molecule that contains a benzyl group, and the structural results showed tranilast located on the hydrophobic region of S100A12. There may be hydrophobic interactions between tranilast and L77 and A79 of the S100A12 protein, which dominated ligand–protein binding (Fig 4D). PROCHECK analysis showed that the HADDOCK data were in a reasonable region because the disallowable region constituted only 1.2% of the total region, and the overall average G-Factor was -0.09 in the usual region (S2 Table).

### 3.4 Fluorescence measurements

**Fig 5:**
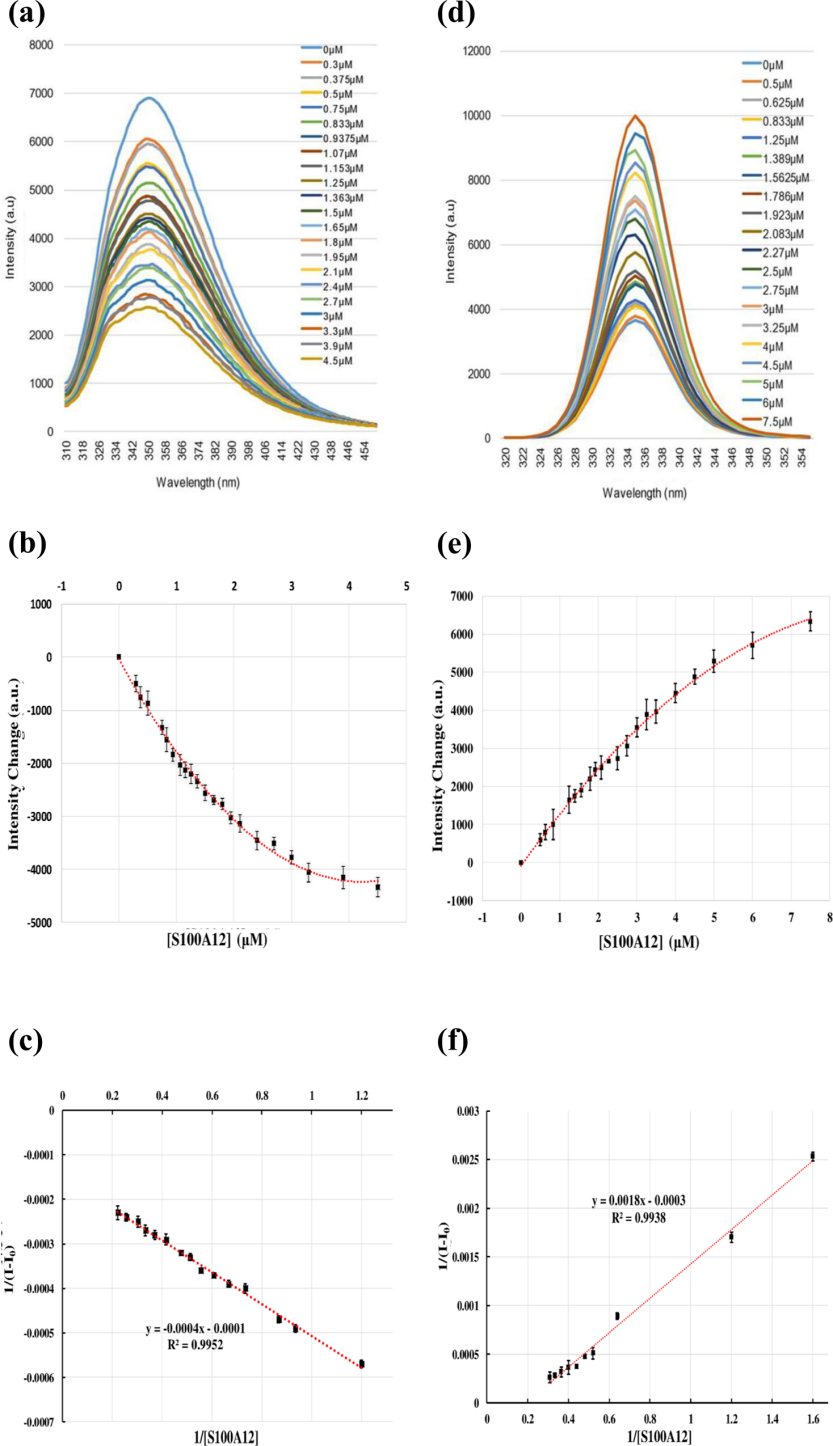
Fluorescence measurements of S100A12 with the RAGE V domain and tranilast. (a) Fluorescence curve of the titration between S100A12 and the RAGE V domain. The initial concentration of the RAGE V domain was 1.5 μM; this was titrated with S100A12 at a concentration of 0–4.5 μM. (b) Curve showing the titration of the RAGE V domain with S100A12 with changes in fluorescence intensity. (c) Linear curve showing the dissociation constant to be 3.1 ± 1.4 μM. The original curve was further processed and calculated using Eq (1). To fit to a linear curve, some outlying points were removed. (d) Fluorescence curve of the titration between S100A12 and tranilast. The initial concentration of tranilast was 2.5 μM; S100A12 was added at a concentration of 0–7.5 μM to measure the emission changes. (e) Curve showing the titration of tranilast with S100A12. (f) Linear curve showing the dissociation constant to be approximately 6.1 ± 1.4 μM. Some points were removed to fit to a linear curve. For all fluorescence experiments, each titration was replicated three times and the error bars are shown on the curves.

There are three tryptophan residues in the RAGE V domain at positions 51, 61, and 72. According to NACCESS analysis, residues 51 and 72 are buried inside the RAGE V domain, and W61 is exposed on the outside of the RAGE V domain. Moreover, W61 is the only tryptophan that is located in the interface region of the RAGE V domain. Therefore, we monitored protein excitation at 295 nm, and a decrease in fluorescence intensity at 350 nm indicated changes in polarity around W61. The charge surrounding W61 became more positive (Fig 3D). The titration curve indicated a decrease in intensity when the S100A12 protein was added to the solution. However, the fluorescence intensity of tranilast increased when the S100A12 protein was added. This result relates to the fluorescence of tranilast, which can absorb at 333 nm; however, the quantum yield of tranilast is very low. It seems that the increase in fluorescence intensity is caused by S100A12 binding with tranilast. The binding of S100A12 and tranilast resulted in less absorption at 333 nm for tranilast. These results were further processed to generate a linear curve. A dissociation constant (K_d_) of approximately 3.1 ± 1.4 μM was calculated for S100A12 binding with RAGE V, and a K_d_ of 6.1 ± 1.4 μM was calculated for S100A12 binding with tranilast. The diagrams and curves of the fluorescence results of S100A12 titrated with the RAGE V domain and S100A12 titrated with tranilast are shown in Fig 5. The results show the binding affinity between S100A12 and the RAGE V domain, which was in the micromolar range, also indicating the stability and formation of the protein complex.

### 3.5 Functional studies of S100A12 with the RAGE V domain and tranilast

**Fig 6:**
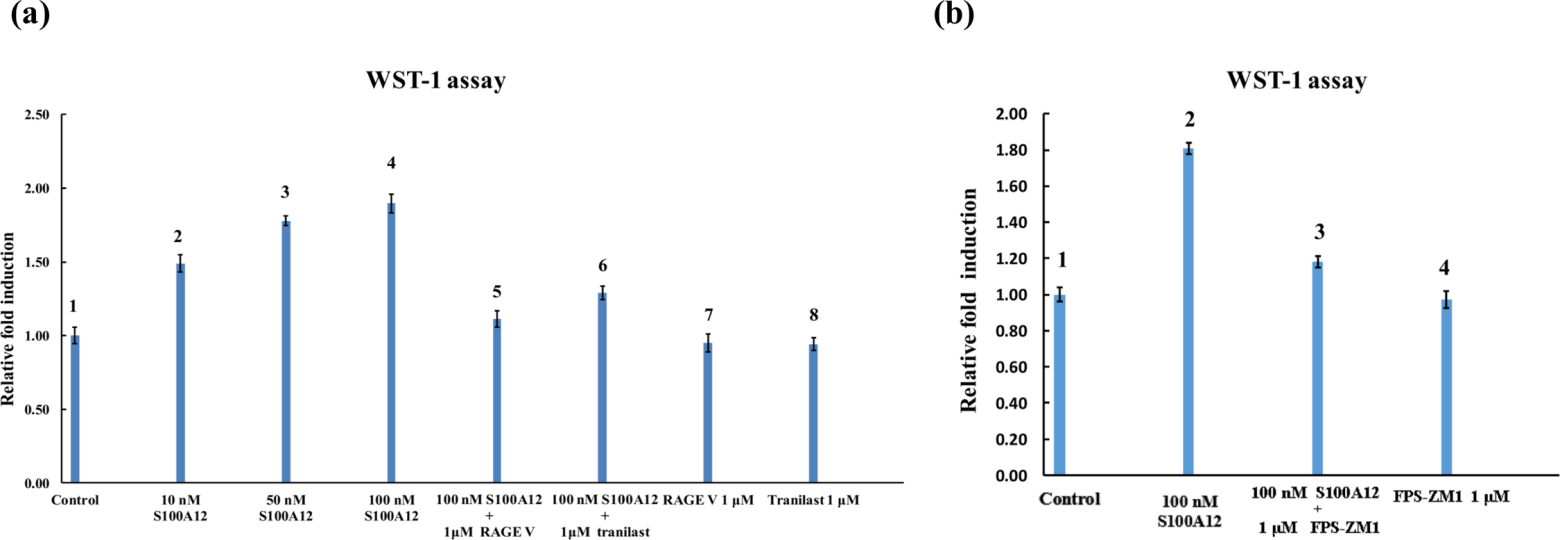
Functional assay of S100A12 with the RAGE V domain and tranilast. (a) SW480 cells were treated with 0 nM (control), 10 nM, 50 nM, or 100 nM S100A12. Cell proliferation was determined after the SW480 cells had been starved of serum for 24 h (lanes 1–4) by adding the WST-1 agent and measuring the optical density. The effects of the other treatments (S100A12 plus 1 μM RAGE V domain and S100A12 plus 1 μM tranilast) on cell proliferation activity were measured for a further 48 h (lanes 5–6). Neither tranilast nor RAGE V domain alone had an effect on cell proliferation activity (lanes 7–8). (b) The serum-starved SW480 cells were treated with 100 nM S100A12, 100 nM S100A12 plus 1 μM FPS-ZM1, or 1 μM FPS-ZM1 for 48 h. The relative cell numbers were determined by WST-1 cell proliferation assay. This experiment was replicated four times and the mean ± standard deviations (SDs) are shown in the plot.

With increasing concentration of S100A12 (10, 50, 100 nM, as shown in Fig 6A, lanes 2, 3, and 4), the SW480 cells grew quickly indicating that the signaling pathway for cell proliferation had been activated by S100A12. Treatment with 1 μM of the RAGE V domain (Fig 6A, lane 5) as a competitor with RAGE on the cell membrane showed an obvious decrease in S100A12-induced cell proliferation activity, whereas treatment with RAGE alone did not alter cell viability. The further treatment of SW480 cells with 1 μM tranilast obviously disrupted S100A12-induced cell proliferation, whereas treatment with tranilast alone had no effect (Fig 6A, lane 6). These results demonstrate that tranilast can effectively block interactions between S100A12 and the RAGE V domain. To determine whether S100A12-induced cell proliferation is mediated by the RAGE pathway, FPS-ZM1 (a RAGE-specific inhibitor), was used to abrogate the interaction between S100A12 and RAGE. The result showed that addition of 1 μM FPS-ZM1 significantly reduced S100A12-induced cell proliferation (Fig 6B, lane 2 and lane 3), suggesting that the signal transduction of cell proliferation requires S100A12 binding to the RAGE V domain on the cell membrane.

## Discussion

RAGE is an important extracellular receptor that can interact with S100 proteins and initiate the transduction of cellular signals. The proteins of the S100 family are known to be important factors in cancer cell proliferation. Several studies on S100 proteins such as S100A6 [38], S100P [39], S100A11 [40], and S100B [64] have been carried out to identify the regions through which they bind to RAGE V. In this study, we have provided insight into the interaction between S100A12 and the RAGE V domain. Using the NMR ^1^H–^15^N HSQC titration method, we showed that interaction takes place through an interface between the hydrophobic region of S100A12 and a loop region of the RAGE V domain.

By comparing with other studies on S100–RAGE, we labeled the S100 protein-binding region in the RAGE V domain structure (PDB code: 2e5e) [48], as shown in the Supporting Information. S100A12 is similar to S100A11, with binding sites at residue 61 to 65, which is a flexible loop. There are hydrophobic interactions between the RAGE V domain and S100A12 (or S100A11). For S100B and mutant S100A6, the binding surface on the RAGE V domain is located at a region with more positive charge, which binds to the negative region of S100B (or mutant S100A6). However, some hydrophobic residues in the RAGE V domain constitute the hydrophobic patch that interacts with the hydrophobic residues in S100B (or mutant S100A6). Similarly, the basic residues (R48, K52, K62, R98, R104, and K110) and hydrophobic residues (L53, G56, W61, V63, P66, G68, P71, and M102) on the surface of the RAGE V domain interact with S100P. However, except for the mutant S100A6–RAGE V complex, the interface between the S100 proteins and the RAGE V domain contains a C-terminal hydrophobic surface at helix 4, which plays an important role in binding with the target proteins. For instance, Y88 and F89 at the C-terminal of S100P play an important role in binding with the cytoskeletal protein ezrin [65]. The actin-capping protein CapZ (TRTK12) interacts with the hydrophobic region-containing helix 4 of S100B [66]. The binding surface between annexin I and S100A11 is also hydrophobic [67]. From the fluorescence experiment, we determined that the dissociation constant was approximately 3.1 ± 1.4 μM for S100A12–RAGE V interaction, which is close to the binding affinity of other S100–RAGE V complexes in the micromolar range.

**Fig 7:**
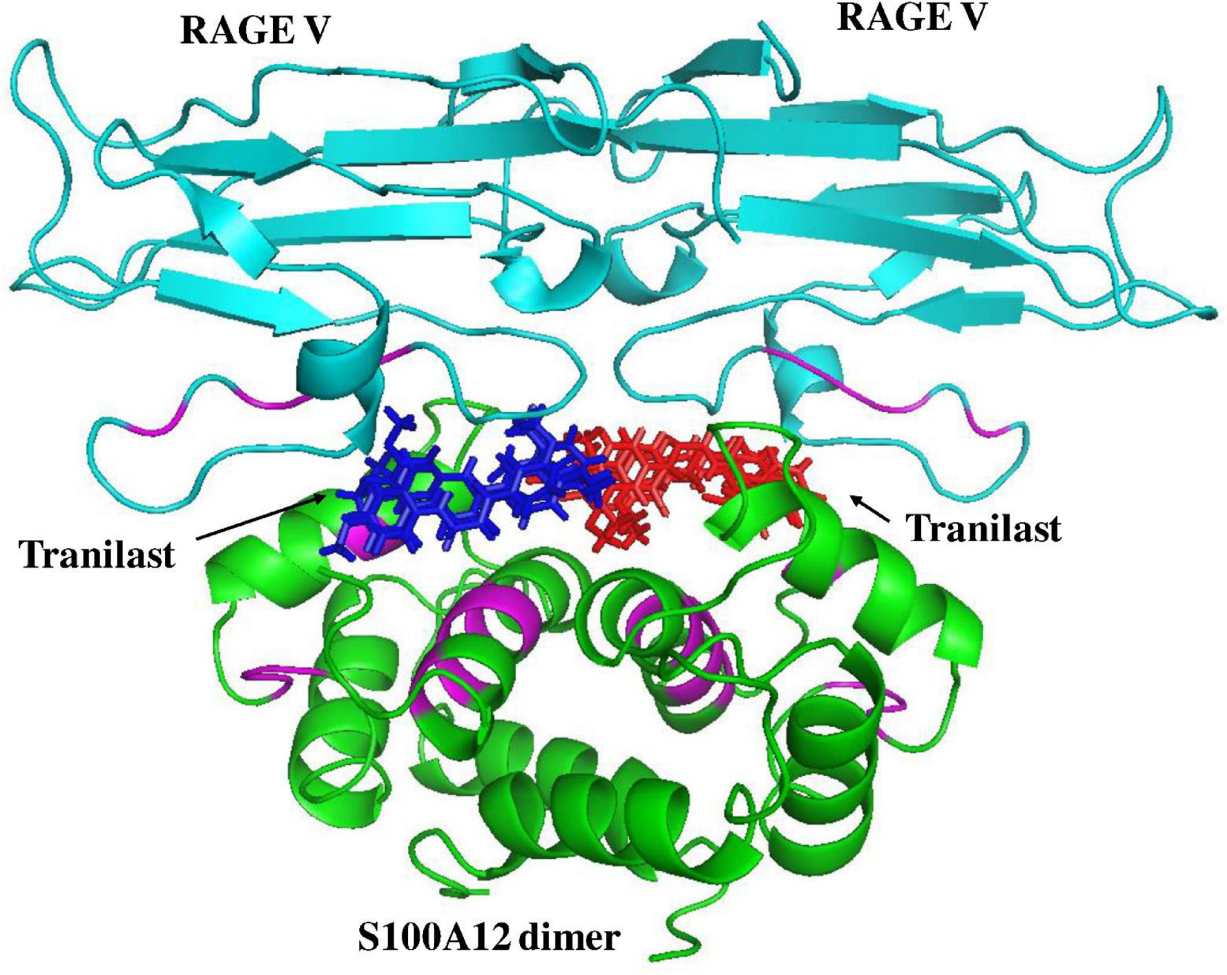
**Overlay of the following two complex structures:** (1) the S100A12 (green) and RAGE V domain (cyan) complex; and (2) the S100A12–tranilast complex (S100A12 is shown in green and tranilast is shown in red and blue). It is clear that tranilast blocks the binding sites (magenta) between S100A12 and the RAGE V domain.

The interaction between S100A12 and RAGE V activates several downstream signaling pathways resulting in different biological responses in cells. For example, in 1999 Hofmann *et al*. [56] reported that S100A12 causes an inflammatory reaction in cells by its interaction with RAGE. Kang *et al*. [68] also showed that S100A12–RAGE binding activated the ERK1/2 and NF-κB signal pathways, resulting in high levels of mucin 5AC (MUC5AC), which is related to chronic obstructive pulmonary disease (COPD). In our current study, tranilast and FPS-ZM1 significantly reduced S100A12-induced cell proliferation (Fig 6A and 6B), suggesting that, at least in part, S100A12-induced cell proliferation takes place through activation of the RAGE pathway. In this study, we further evaluated a useful drug, tranilast, and used ^1^H–^15^N HSQC titration to show that it interacts with S100A12. As shown in Fig 7, we overlaid the two complex structures obtained in this study: (1) the S100A12 and RAGE V domain complex (shown in cyan); and (2) the S100A12 (green) and tranilast (red and blue) complex. This figure clearly shows that tranilast can block binding between S100A12 and the RAGE V domain.

Moreover, using fluorescence titration measurement we obtained the dissociation constants for both S100A12–RAGE and S100A12–tranilast. Although the K_d_ of tranilast was lower than that of RAGE V with S100A12, it still represents a potential precursor for the development of new inhibitors. Further evaluation using the WST-1 assay indicated that tranilast efficiently inhibited cell proliferation *in vitro*. Our results have provided structural insight into the S100A12–RAGE V complex and identified a precursor for the development of new drugs. These results may be useful for the generation of therapies that focus on RAGE and S100 protein-related diseases.

## Supporting Information

S1 Fig Molecular structure of tranilast. (TIF)

S2 Fig Analysis by electrospray ionization time-of-flight mass spectrometry (ESI-TOF-MS) to confirm the molecular weight of S100A12. (TIF)

S3 Fig Analysis by electrospray ionization time-of-flight mass spectrometry (ESI-TOF-MS) to confirm the molecular weight of the RAGE V domain. The molecular weight difference was approximately 2.6 Da owing to a disulfide bond inside the V domain of RAGE.
(TIF)

S4 Fig Analysis of the ^1^H–^15^N HSQC spectra of the labeled RAGE V domain with tranilast. Overlay of the ^1^H–^15^N HSQC spectra of 0.5 mM ^15^N-labeled RAGE V domain (red) and RAGE V domain titrated with 0.5 mM tranilast (green).
(TIF)

S5 Fig Replications of the fluorescence titration experiment on S100A12–tranilast. We replicated the experiment with 2.5 μM tranilast and titrated with S100A12 protein. We colored the linear curve in a different color for each replication. The dissociation constant was approximately 6.1 ± 1.4 μM.
(TIF)

S6 Fig Replications of the fluorescence titration experiment on S100A12–RAGE V domain. We replicated the experiment with the 1.5 μM RAGE V domain and titrated with S100A12 protein. We colored the linear curve in different colors for each replication. The dissociation constant was approximately 3.1 ± 1.4 μM.
(TIF)

S7 Fig Binding interface of RAGE V domain with different S100 proteins. We used the RAGE V domain from the Protein Data Bank (PDB code: 2e5e) and labeled the binding sites with different colors (red: S100A12; blue: S100A11; purple: mutant S100A6; and yellow: S100P).
(TIF)

S1 File Expression and purification of S100A12 and the V domain of RAGE. (DOCX)

S1 Table Analysis of the S100A12–RAGE V domain by PROCHECK. The picture shows the rationalization of the residues in the complex structure and the data indicate the percentage of allowed and disallowed regions. Only 1.2% (4 residues) were disallowed. Furthermore, the G-Factors also indicate that the result is a reasonable region because the overall average was only 0.1 (larger than -0.5, which signifies an unusual result).
(TIF)

S2 Table Analysis of the S100A12–tranilast complex by PROCHECK. The diagram was constructed on the PROCHECK website. The picture shows the rationalization of the residues in the complex structure and the data indicate the percentage of allowed and disallowed regions. Only 1.24% (2 residues) were disallowed. Furthermore, the G-Factors also indicate that the result is a reasonable region because the overall average was only -0.09 (larger than -0.5, which signifies an unusual result).
(TIF)

## Abbreviations

S100A12: human S100 calcium-bound protein
RAGE: the receptor for advanced glycation end products
HSQC: heteronuclear single quantum correlation
HADDOCK: high ambiguity driven docking
DTT: dithiothreitol
E. coli: *Escherichia coli*
OD: optical density
IPTG: isopropyl β-D-thiogalactopyranoside
SDS-PAGE: sodium dodecyl sulfate polyacrylamide gel electrophoresis
