# Supplementary figures and images for: Blocking the Interactions between Calcium-Bound S100A12 Protein and the V Domain of RAGE Using Tranilast
Source: PLoS One. 2016 Sep 6;11(9):e0162000. doi: 10.1371/journal.pone.0162000 (PMC5012620; doi:10.1371/journal.pone.0162000)

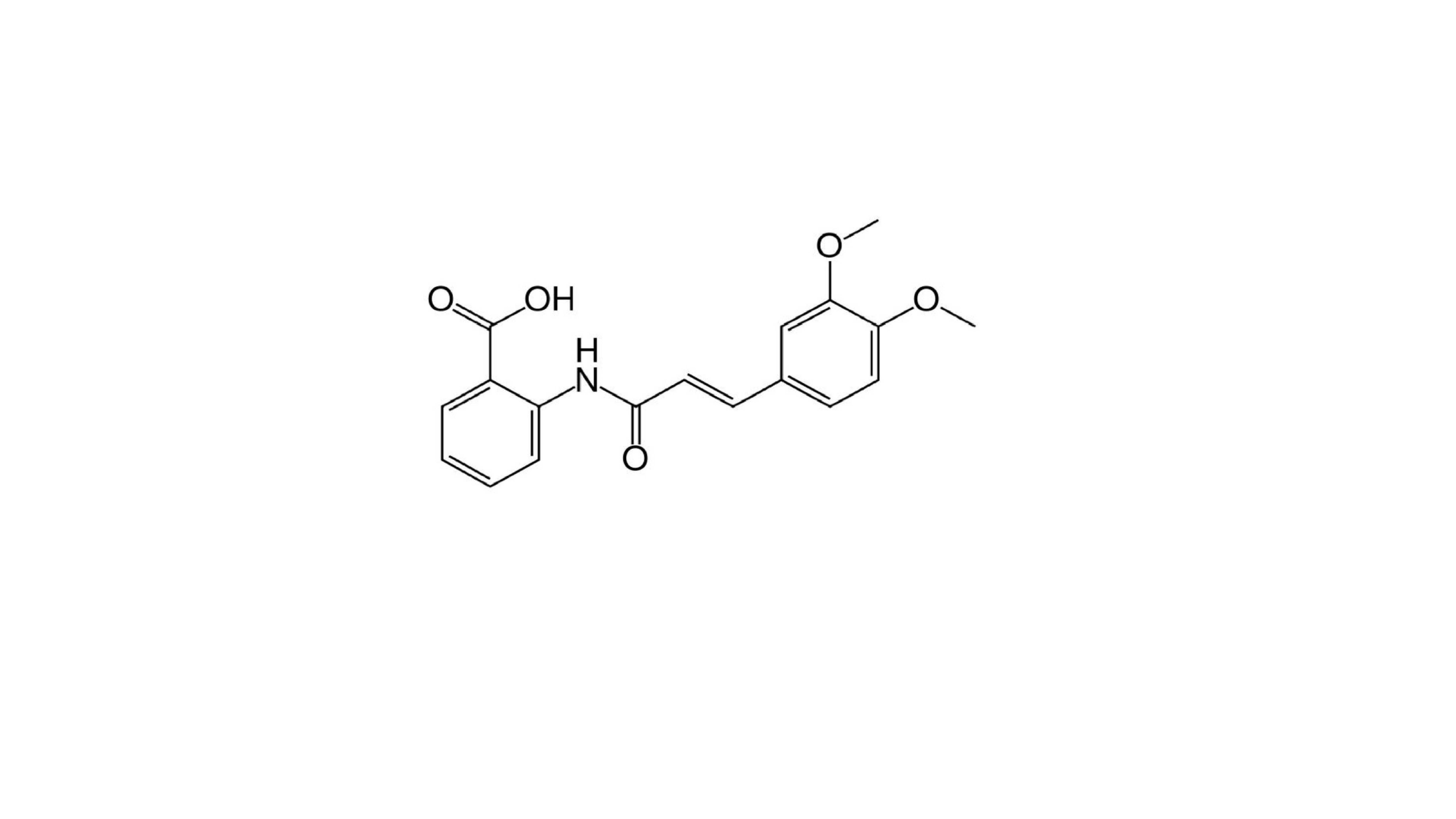

Supplement: S1 Fig — (TIF) [file pone.0162000.s001.tif]

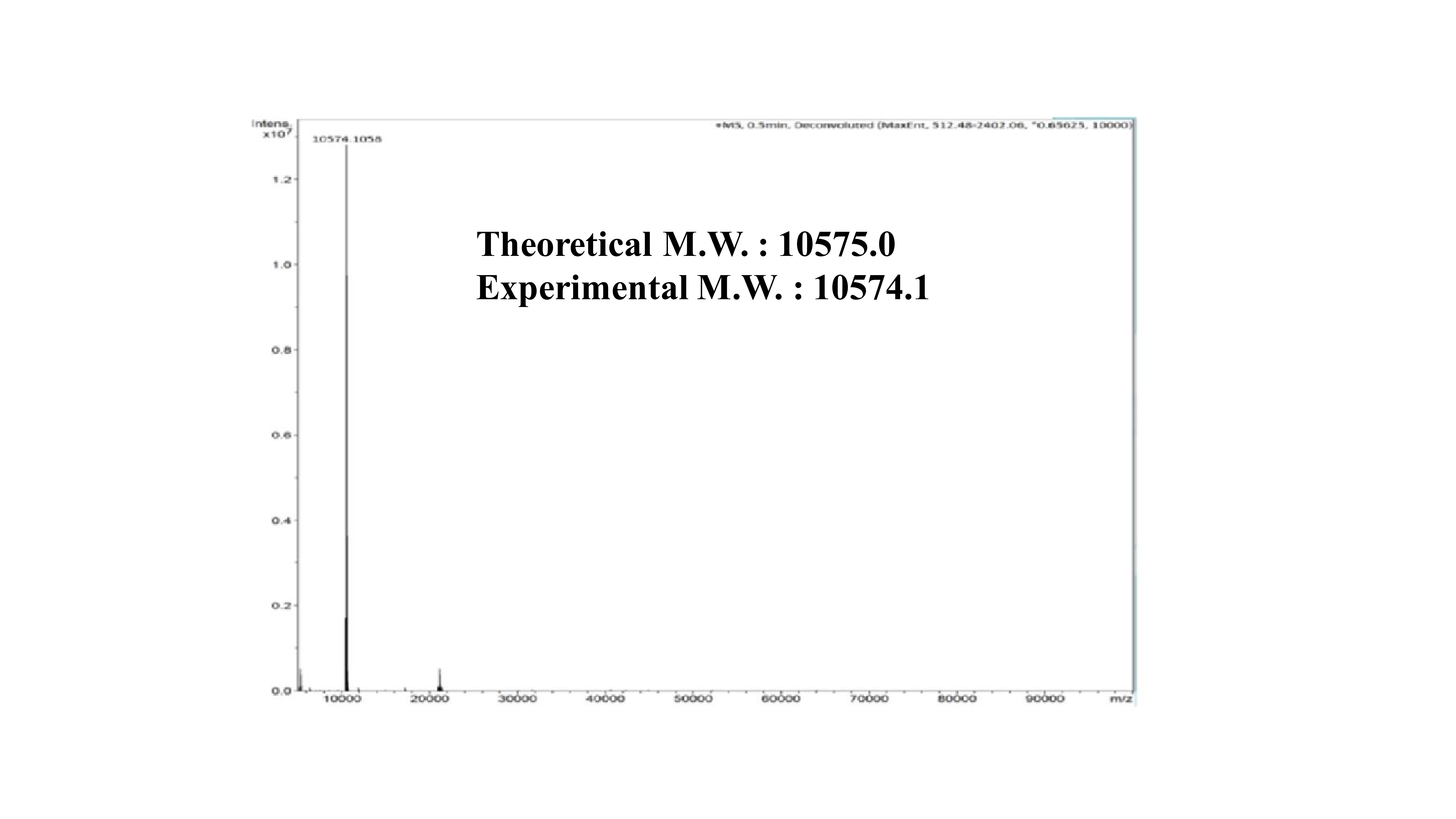

Supplement: S2 Fig — (TIF) [file pone.0162000.s002.tif]

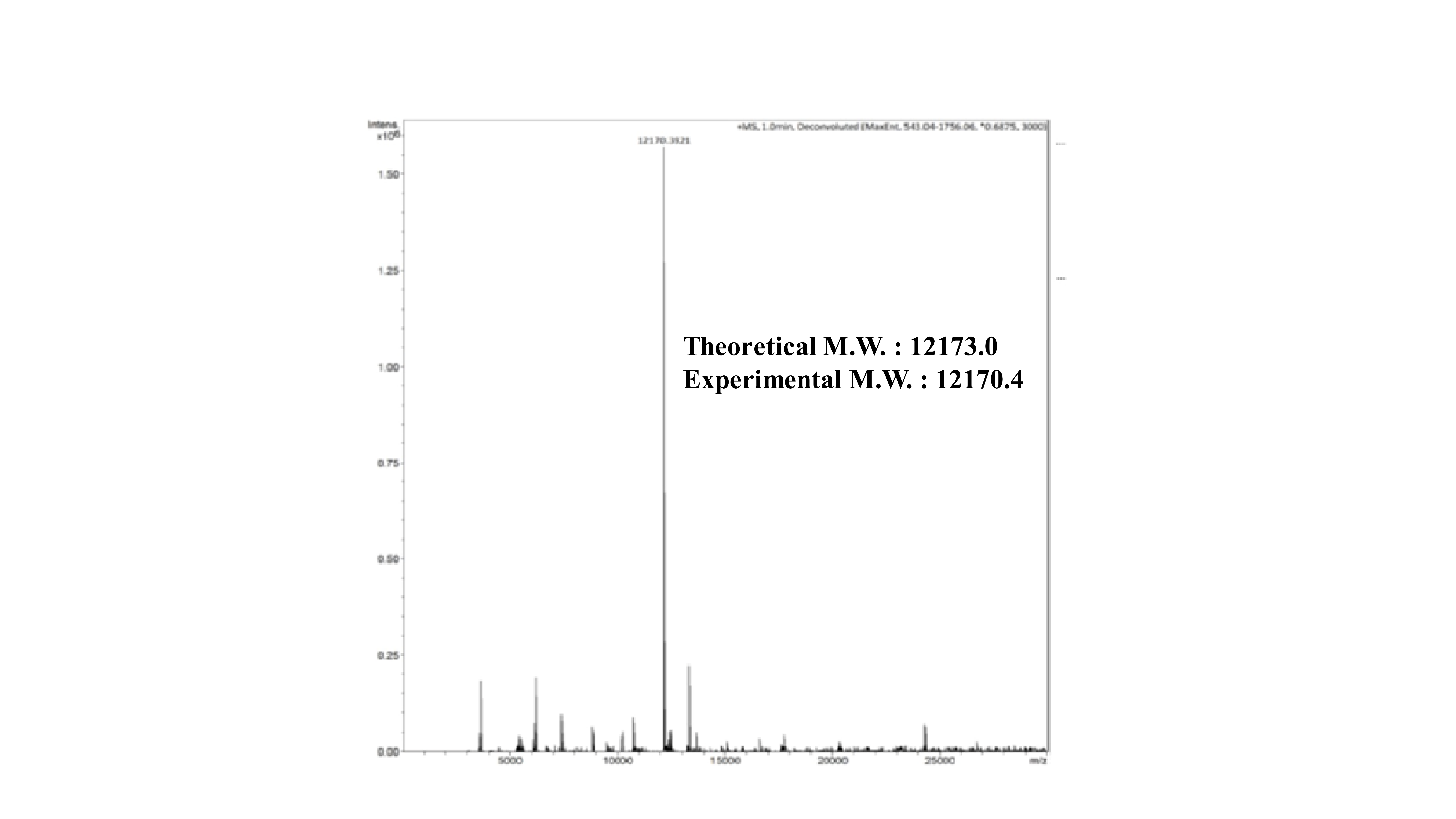

Supplement: S3 Fig — The molecular weight difference was approximately 2.6 Da owing to a disulfide bond inside the V domain of RAGE. (TIF) [file pone.0162000.s003.tif]

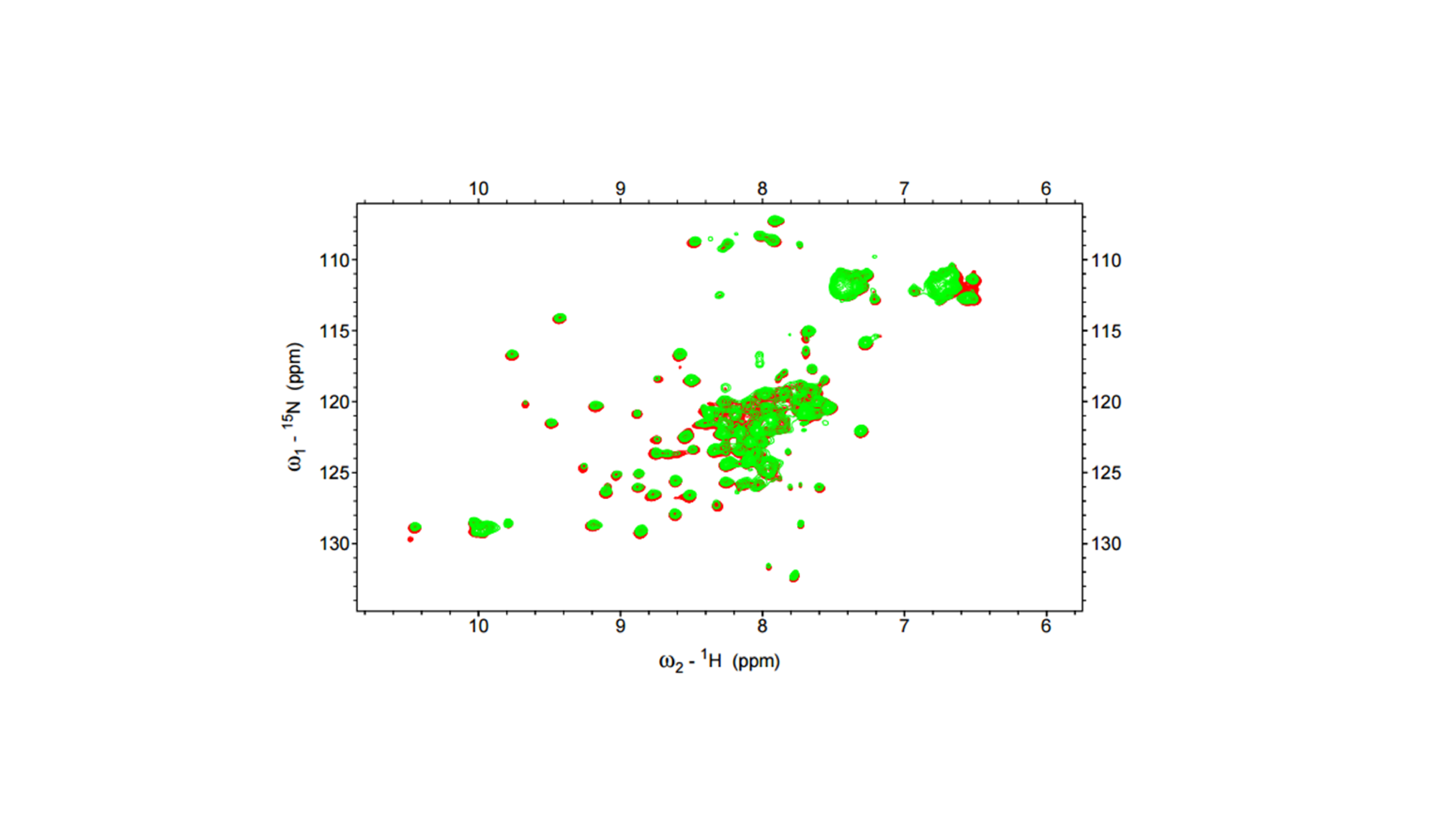

Supplement: S4 Fig — Overlay of the 1H–15N HSQC spectra of 0.5 mM 15N-labeled RAGE V domain (red) and RAGE V domain titrated with 0.5 mM tranilast (green). (TIF) [file pone.0162000.s004.tif]

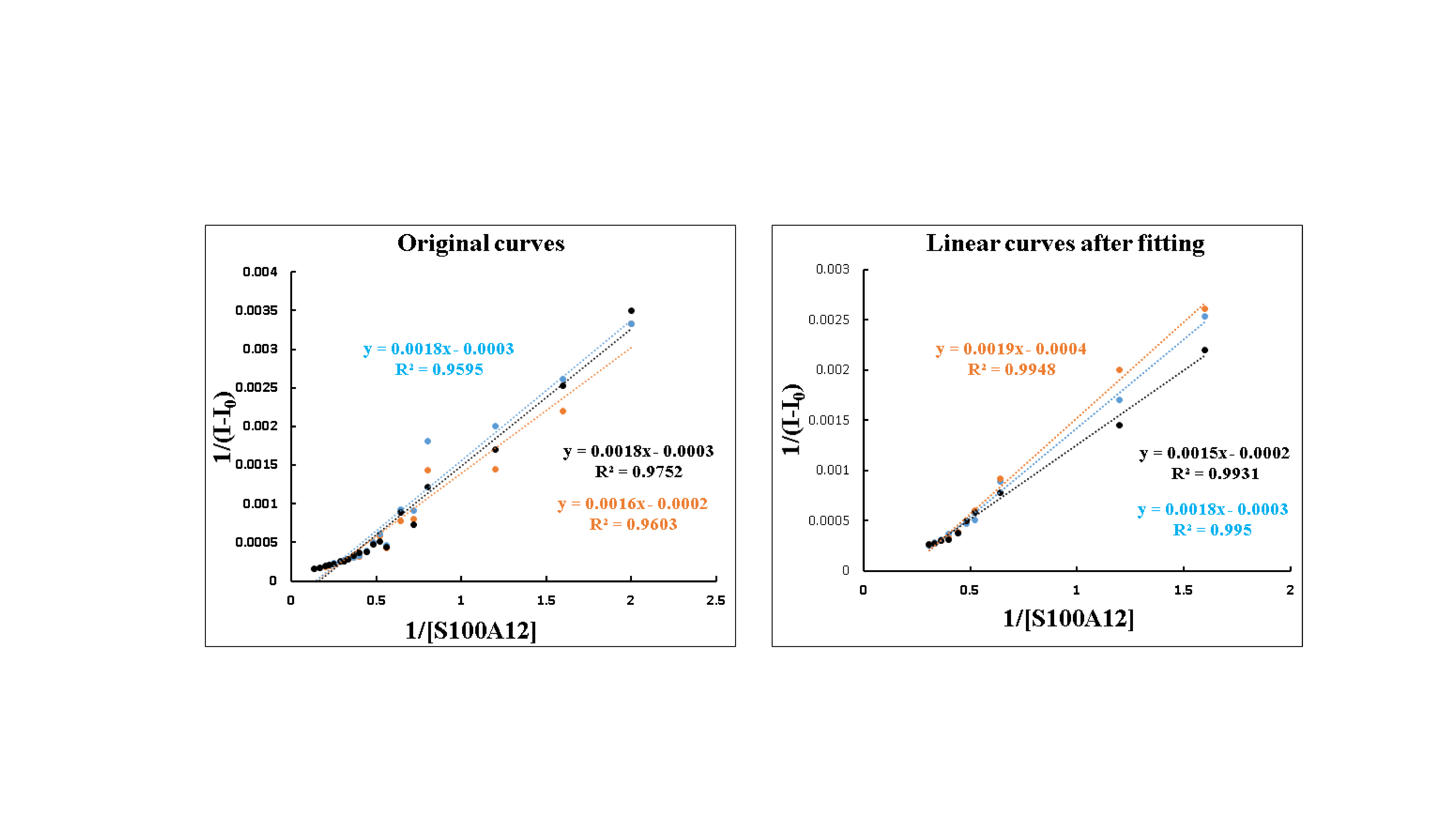

Supplement: S5 Fig — We replicated the experiment with 2.5 μM tranilast and titrated with S100A12 protein. We colored the linear curve in a different color for each replication. The dissociation constant was approximately 6.1 ± 1.4 μM. (TIF) [file pone.0162000.s005.tif]

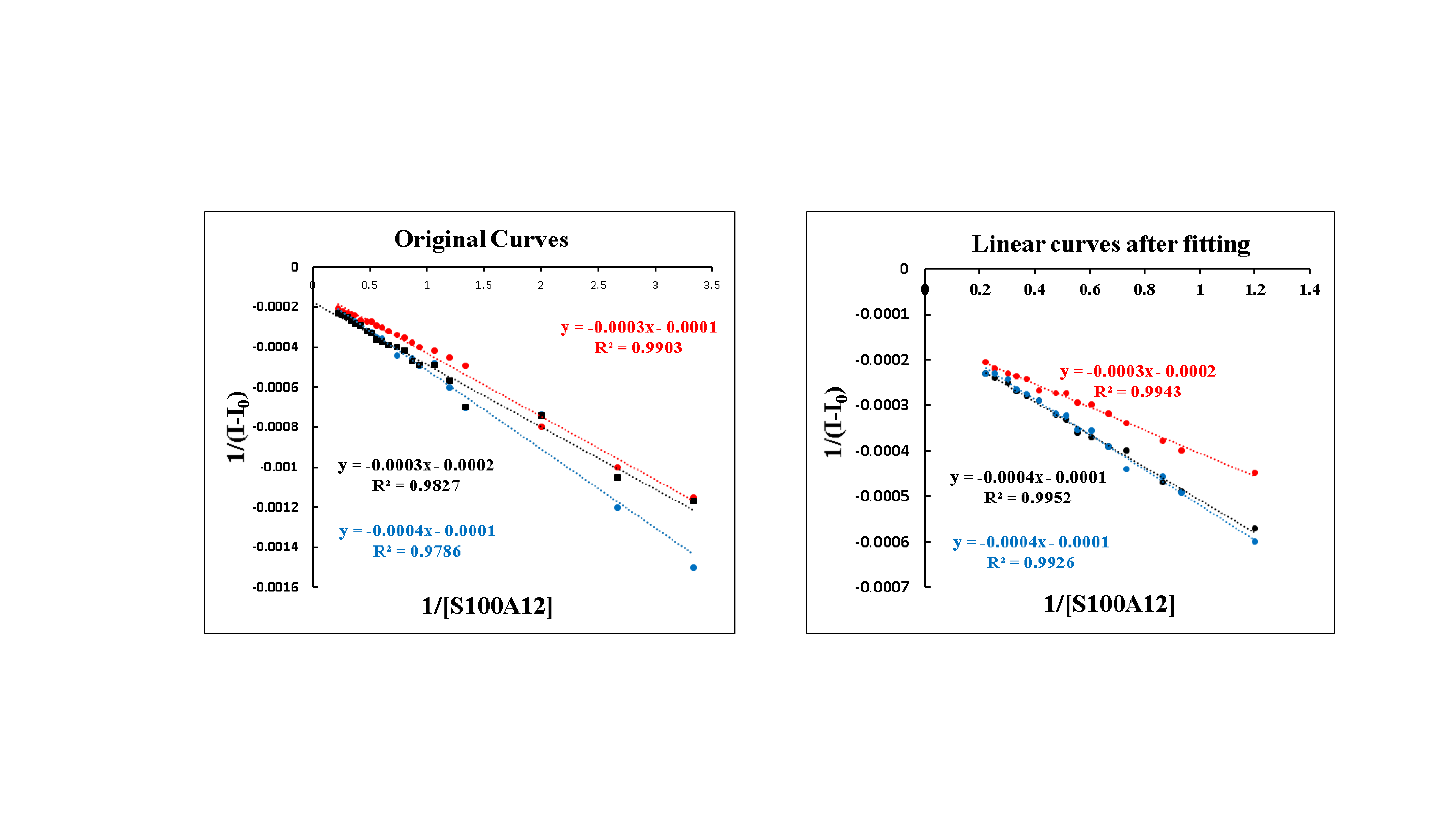

Supplement: S6 Fig — We replicated the experiment with the 1.5 μM RAGE V domain and titrated with S100A12 protein. We colored the linear curve in different colors for each replication. The dissociation constant was approximately 3.1 ± 1.4 μM. (TIF) [file pone.0162000.s006.tif]

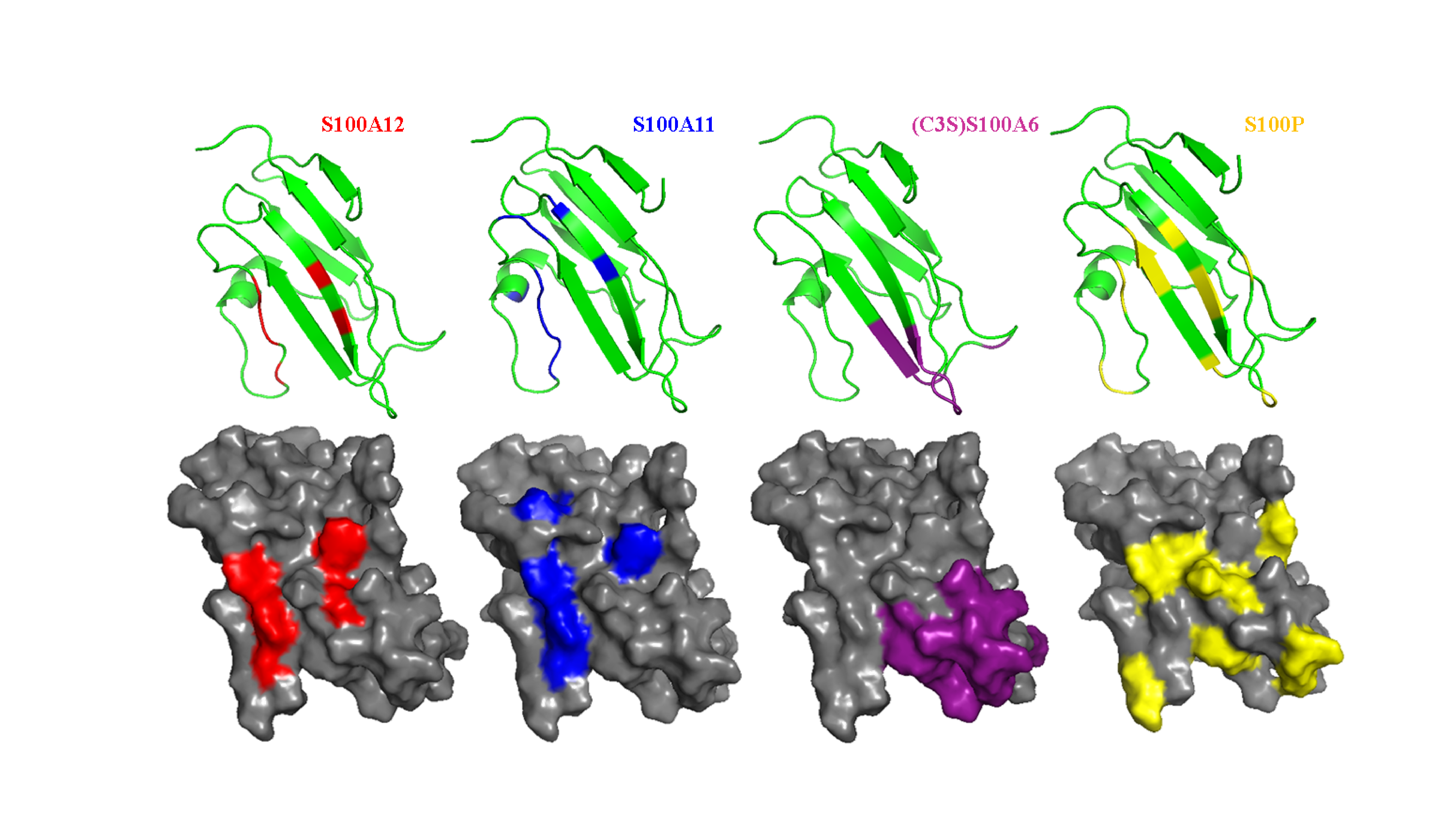

Supplement: S7 Fig — We used the RAGE V domain from the Protein Data Bank (PDB code: 2e5e) and labeled the binding sites with different colors (red: S100A12; blue: S100A11; purple: mutant S100A6; and yellow: S100P). (TIF) [file pone.0162000.s007.tif]

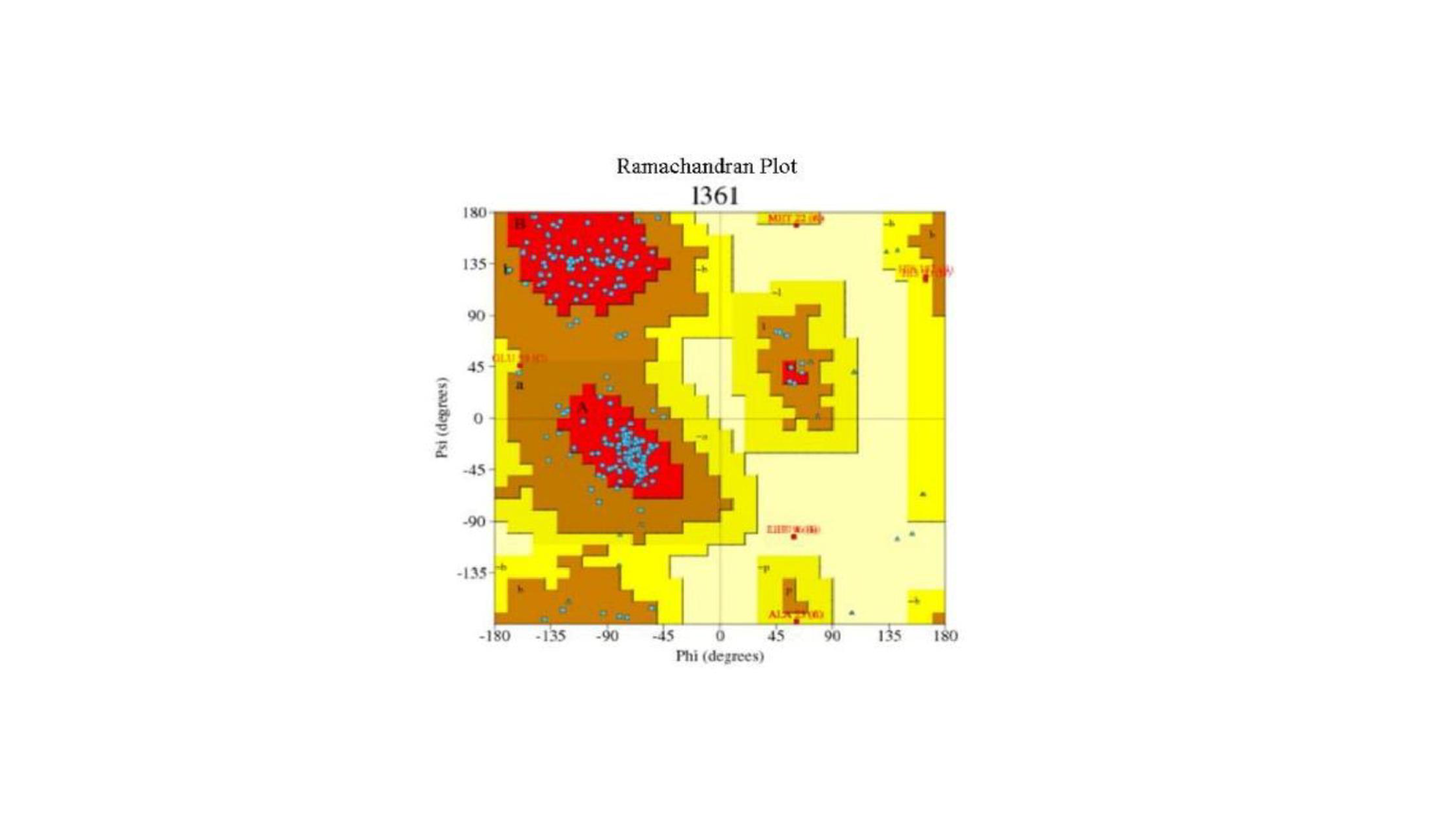

Supplement: S1 Table — The picture shows the rationalization of the residues in the complex structure and the data indicate the percentage of allowed and disallowed regions. Only 1.2% (4 residues) were disallowed. Furthermore, the G-Factors also indicate that the result is a reasonable region because the overall average was only 0.1 (larger than -0.5, which signifies an unusual result). (TIF) [file pone.0162000.s009.tif]

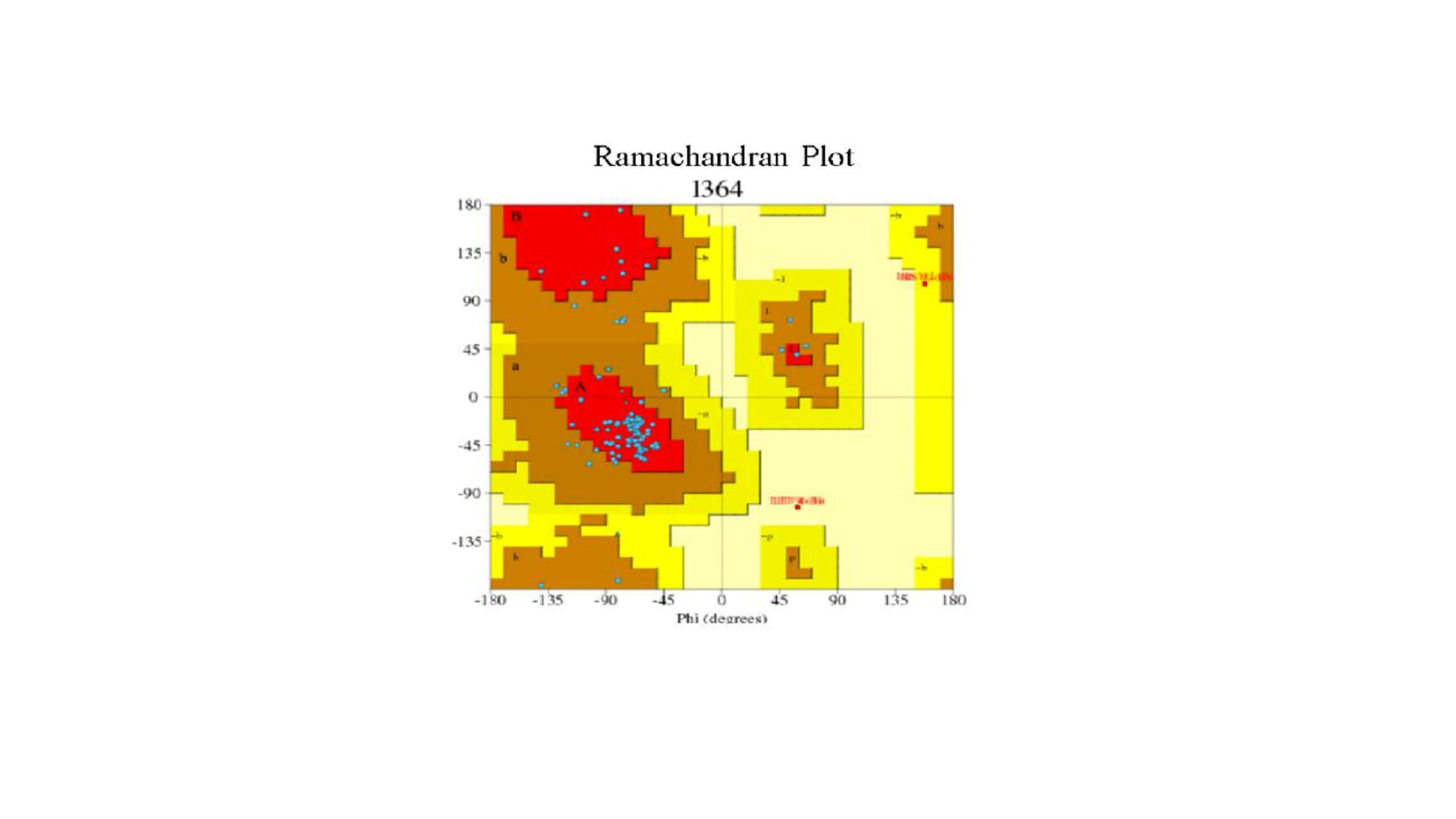

Supplement: S2 Table — The diagram was constructed on the PROCHECK website. The picture shows the rationalization of the residues in the complex structure and the data indicate the percentage of allowed and disallowed regions. Only 1.24% (2 residues) were disallowed. Furthermore, the G-Factors also indicate that the result is a reasonable region because the overall average was only -0.09 (larger than -0.5, which signifies an unusual result). (TIF) [file pone.0162000.s010.tif]
