# Supplementary material for: Blocking the Interactions between Calcium-Bound S100A12 Protein and the V Domain of RAGE Using Tranilast
Source: PLoS One. 2016 Sep 6;11(9):e0162000. doi: 10.1371/journal.pone.0162000 (PMC5012620; doi:10.1371/journal.pone.0162000)
Supplement: S1 File — (DOCX) [file pone.0162000.s008.docx]

**Expression and purification of S100A12 and the V domain of RAGE**

The overexpressed samples were grown in M9 medium using ^15^N-ammonium chloride as the sole nitrogen source for preparing the labeled sample. For the unlabeled sample, cells were grown in Lysogeny broth (LB) medium. The bacterial culture was grown until the optical density (OD) at 600 nm reached 0.7. Protein expression was induced by adding 1 mM isopropyl β-D-thiogalactopyranoside (IPTG) at 298 K. After 16 h of induction, the cells were centrifuged at 6,000 rpm for 25 min at 277 K, and then the cell pellets were suspended in 20 mL of buffer (20 mM Tris-HCl, pH 8). We lysed the cells using a French press, and the cell debris was separated by centrifugation at 12,000 rpm at 277 K for 1 h. S100A12 was present in the soluble fraction after centrifugation. We collected the supernatant fraction and purified it using a Hi-Prep Q XL 16/10 column. After the supernatant had passed through, we washed the column with equilibrium buffer (20 mM Tris-HCl, pH 8) and eluted the protein with elution buffer (20 mM Tris-HCl, 1 M NaCl, pH 8). The S100A12-containing fraction was collected and dialyzed against a buffer (50 mM Tris-HCl, 5 mM CaCl_2_, 150 mM NaCl, pH 7.5) for further purification using a Hi-Prep Phenyl FF 16/10 column. First, we used the loading buffer (50 mM Tris-HCl, 5 mM CaCl_2_, 150 mM NaCl, pH 7.5) to balance the column and injected the previous sample containing S100A12 into the column. Next, we washed the column with loading buffer (50 mM Tris-HCl, 5 mM CaCl_2_, 150 mM NaCl, pH 7.5) to remove impurities. Finally, we eluted the target protein with elution buffer (50 mM Tris-HCl, 5 mM ethylenediaminetetraacetic acid (EDTA), 100 mM NaCl, pH 7.5). The S100A12-containing fraction was concentrated and dialyzed against the NMR buffer (20 mM Tris-HCl, 5 mM CaCl_2_, 100 mM NaCl, pH 7).

The recombinant cDNA of the RAGE V domain (residues 22–121) was subcloned into the pET-32b (+) vector, which was purchased from Mission Biotech Company. The overexpressed cells were grown in M9 or LB medium until the OD at 600 nm reached 0.7. Then, 1 mM IPTG was added to induce protein expression at 298 K. After 3 h of induction, cells were centrifuged at 6,000 rpm at 277 K, and the pellets were suspended in 20 mL buffer (20 mM Tris-HCl, 300 mM NaCl, pH 8). To obtain the protein, we used a French press to lyse the cells, and the cell debris was removed by centrifugation at 12,000 rpm for 1 h. The proteins remained in the supernatant fraction, which was collected for purification by nickel column chromatography. The RAGE V domain had a six-histidine tag at the N-terminus that had high affinity with the nickel column. For purification, we washed and balanced the nickel column with equilibrium buffer (20 mM Tris-HCl, 300 mM NaCl, pH 8) before the protein was passed through the column. Next, we washed out the impurities with buffer (20 mM Tris-HCl, 300 mM NaCl, 20 mM imidazole, pH 8). Finally, we used elution buffer (20 mM Tris-HCl, 300 mM NaCl, 300 mM imidazole, pH 8) and collected the eluted fraction for concentration. After concentration, we added approximately 45 units of thrombin to cleave the tag at the N-terminus, and incubated the RAGE V domain in a water bath at 298 K for 3 h. Finally, we used high-performance liquid chromatography (HPLC) to obtain the pure RAGE V domain and changed the buffer to NMR buffer. The purity of the sample was estimated to be approximately 95% by Coomassie-stained SDS-PAGE and HPLC analyses. All molecular weights were confirmed by electrospray ionization time-of-flight mass spectrometry (ESI-TOF-MS).
